# CellProfiler Analyst: data exploration and analysis software for complex image-based screens

**DOI:** 10.1186/1471-2105-9-482

**Published:** 2008-11-15

**Authors:** Thouis R Jones, In Han Kang, Douglas B Wheeler, Robert A Lindquist, Adam Papallo, David M Sabatini, Polina Golland, Anne E Carpenter

**Affiliations:** 1 Imaging Platform, Broad Institute of MIT and Harvard, Cambridge, MA 02142, USA; 2 Whitehead Institute for Biomedical Research, Cambridge, MA 02142, USA; 3 Computer Sciences and Artificial Intelligence Laboratory, Massachusetts Institute of Technology, Cambridge, MA 02142, USA; 4 Department of Biology, Massachusetts Institute of Technology, Cambridge, MA 02142, USA

## Abstract

**Background:**

Image-based screens can produce hundreds of measured features for each of hundreds of millions of individual cells in a single experiment.

**Results:**

Here, we describe CellProfiler Analyst, open-source software for the interactive exploration and analysis of multidimensional data, particularly data from high-throughput, image-based experiments.

**Conclusion:**

The system enables interactive data exploration for image-based screens and automated scoring of complex phenotypes that require combinations of multiple measured features per cell.

## Background

Visual analysis of cell samples has played a dominant role in the history of biology. The scientific community has only begun to scratch the surface of computationally extracting the rich information visible in fluorescence microscopy images of cell samples [1]. This capability is increasingly important given the ease now to systematically perturb cells with libraries of chemicals or gene-perturbing reagents like RNA interference or gene overexpression and collect hundreds of thousands of images of these cell samples [2,3]. We recently developed open-source image analysis software, CellProfiler, which measures a rich set of cellular features in images, such as size, shape, and staining patterns including intensity, texture, and colocalization [4,5]. This tool has been useful for extracting image-based measurements to score sophisticated screens [6-8], with many more in progress.

The volume and richness of individual-cell data from large image-based screens is unprecedented and existing software is inadequate for the challenge of data analysis. For analysis of small or very simple experiments, spreadsheet programs like Microsoft Excel are sufficient, and useful open-source tools exist for analysis and exploration of data from high throughput screens in general [9-12]. Existing software packages targeted for image-based screening, however, have one or more limitations which prevent sophisticated visualization and extraction of information from image-based screens: (a) they are not designed for the hierarchical data structure inherent in image-based data (each treatment condition is replicated in several samples, each sample is usually represented by several images, each image contains a population of cells, and each cell has hundreds of associated measures), (b) they ignore the inherent biological variability of cell populations such that assays requiring subpopulation analysis cannot be scored, (c) they cannot handle the volumes of data typical in image-based experiments (e.g., ~500 measurements for each of ~100 million individual cells), (d) they provide limited linking to raw or processed image data or chemical structure data, (e) they allow only limited statistical analyses of the data, (f) they are proprietary and new methods cannot be easily added, (g) they are limited to data from a particular image analysis package, (h) they require expertise in statistics or programming, and/or (i) they require intense hands-on data management.

Given that no existing tools meet the specific needs of image-based screens, researchers have needed computational expertise to directly query databases of image-based information using command-line tools. Often, the researchers best able to explore and interpret the data lack these computational skills. These researchers are therefore less likely to make serendipitous discoveries (or identify quality-control issues) in their image-based screens, which inherently contain enormous amounts of information beyond that which is pertinent to the original, intended biological question. It is critical to provide exploration tools to screening researchers, tools that employ their understanding of the experiment in question and their creativity and ability to recognize and interpret patterns and relationships within data. These capabilities flourish when united with a computer's unique ability to store, retrieve, display, and quantitatively analyze billions of data points.

We therefore sought to develop a software system that would make high-dimensional image-based data exploration feasible for researchers who lack computational skills, and flexible for computer scientists who want to develop and add advanced new methods for image-based screening, such as machine learning-based phenotype scoring. We describe here the result of our work, an open-source software package called "CellProfiler Analyst".

## Results and discussion

### Viewing data

**Figure 1:**
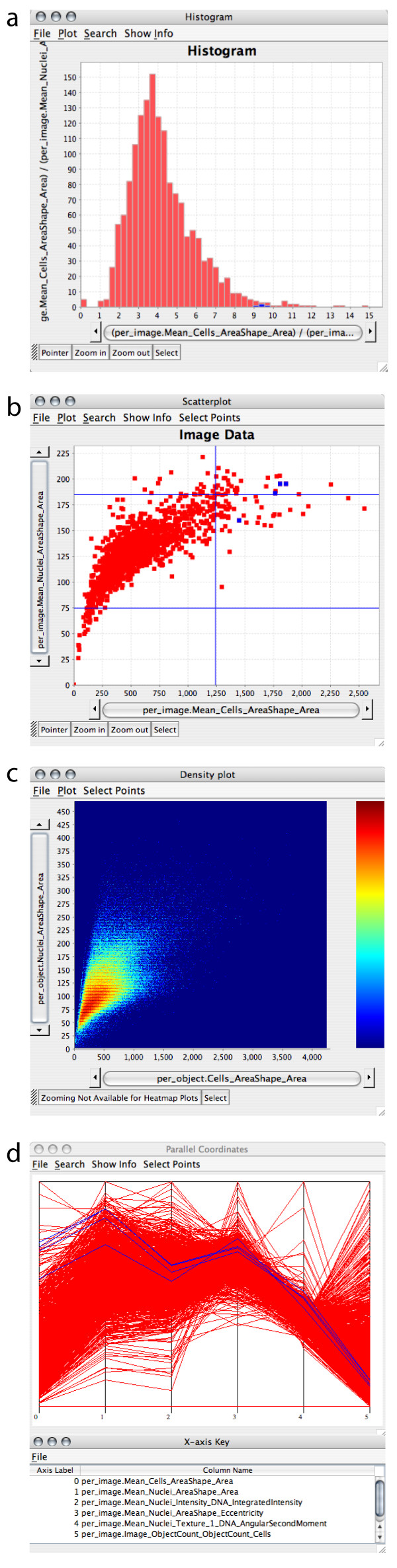
**Four types of plots can be created**. Four types of plots created by CellProfiler Analyst are shown. (a) A histogram of per-image data (the mean area of all cells in the image). (b) Scatterplot of image data (X-axis = mean area of all cells in the image; Y-axis = mean area of all nuclei in the image). One particular sample and its replicates are highlighted as blue data points, and the blue lines indicate +/- two standard deviations from the mean, although because the data is non-Gaussian, the left standard deviation line is not visible. (c) Density plot of individual cell data (X-axis = area of the cell; Y-axis = area of the nucleus. (d) Parallel coordinate plot where 6 features are plotted, one on each numbered axis as labeled in the table shown below the plot. The four selected blue data points in the scatterplot are also highlighted in the parallel coordinate plot as blue lines. From this plot, it is apparent that these four data points have high cell and nuclear area (two left-most coordinates) but low cell count (right-most coordinate).

Four types of plots are the starting points for exploration of large, multi-dimensional image-based screens in CellProfiler Analyst (Figure 1). Importantly, these tools are compatible with the scale of data typically acquired in image-based screens, which can be hundreds of features for each of hundreds of millions of cells. Histograms display the distribution of values for one measured feature by grouping image or object data into evenly spaced bins, on a linear or logarithmic scale (Figure 1a). Such plots can be helpful, for example, to examine the cell cycle status of samples (by plotting per-cell DNA content) or to examine outliers for quality control purposes (e.g., by plotting per-image cell counts). Two measured features per image or object can be displayed on the same chart via a scatterplot (Figure 1b), which is also useful for identifying hits and for quality control purposes. For example, the researcher can readily exclude out-of-focus images from analysis based on measurements made by CellProfiler's "Measure Image Quality" module. Because data points in scatterplots can occlude each other, they are typically unsuitable for individual cell data where hundreds of millions of data points are examined to identify interesting subpopulations. For these cases, a density plot is more appropriate (Figure 1c). Every pixel in the plot represents a histogram "bin" and the color of the pixel represents the number of data points in the bin. These plots are useful, for example, for establishing thresholds at which to classify individual cells as "positive" or "negative" based on two features (e.g., based on two intensity measures as in flow cytometry). To explore more than two measured features of each image or data point, a parallel coordinate plot is used. Parallel coordinate plots [13] allow analysis of multiple dimensions of data, whereby each measured feature's scaled (0–1) values are given a separate y-axis and individual data points are connected across these multiple axes (Figure 1d).

Each data point in a plot can represent an individual cell or, by contrast, the mean value of the population of cells within an image. Data can also be grouped by characteristics the samples have in common (e.g., chemical name or dose). Multiple experiments that investigate the same set of treatment conditions (e.g., chemical compounds or RNA interference reagents) can be grouped together, which eases analysis of replicates. For all types of plots, the data to be displayed can be filtered, for example to plot data only from a single image, from a sample of data points at specified equal intervals, or data that satisfies certain criteria (specified in SQL "where" clauses like "CellCount > 100").

### Exploring relationships among data

**Figure 2:**
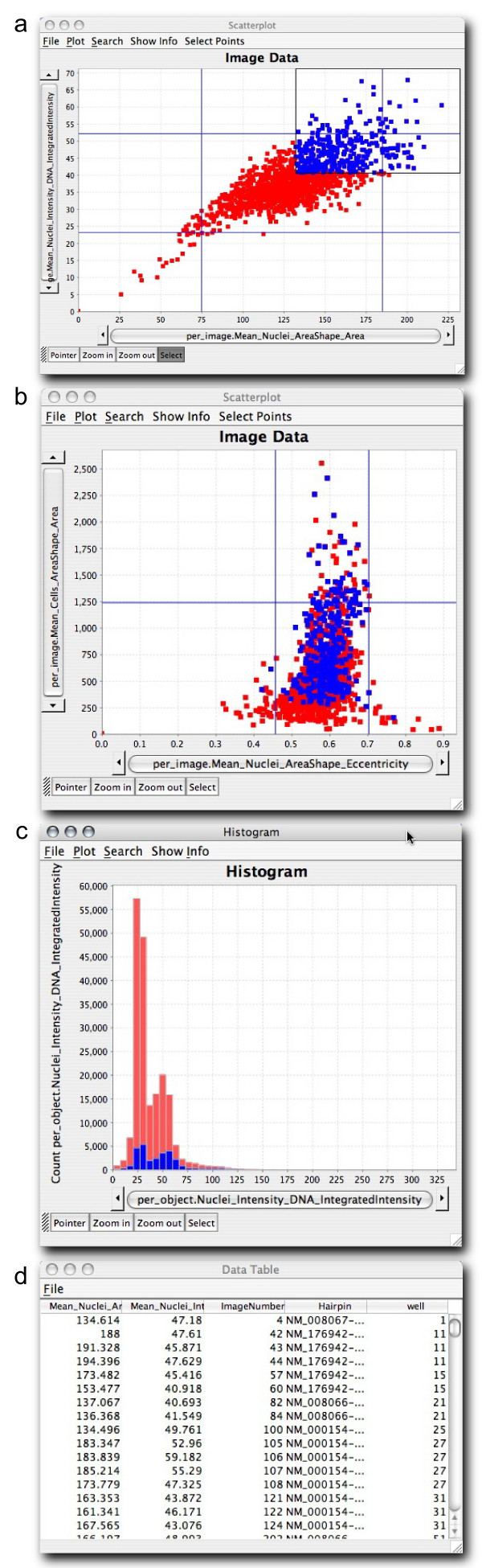
**Relationships among data can be explored**. Data points representing images with high nuclear area (averaged over the cells in the image) and high DNA intensity (averaged over the cells in the image) are highlighted in blue by brushing the scatterplot shown in (a). Immediately, the corresponding points appear blue in the other open scatterplot (b), allowing the relationship between all of the plotted features to be examined. As well, a DNA content histogram (c) shows individual cell data from the selected image data points (blue), relative to all cells in the experiment (red). In this case, the selected blue data points indeed have an unusual cell cycle distribution (fewer 2N cells relative to 4N cells) as compared to all cells in the experiment. Finally, the sample names (d, "Hairpin" column, for this particular RNA interference experiment) corresponding to the selected blue data points can be displayed in a table to see which samples are present in the selected points. The first two columns of the table show other information about those data points based on the axes of the scatterplot shown in (a). The "ImageNumber" and "well" columns provide additional information about the samples the researcher is investigating.

Data points selected and highlighted in one plot are immediately highlighted in all other open plots (a technique often called "brushing" [14]) such that a sample or set of samples can be examined in the context of other sets of samples (Figure 2). This allows, for example, the comparison of measurements from samples of interest vs. all samples in the experiment. Brushing helps the user to more easily examine relationships in the data, especially when the data has a large number of attributes or items, when the data spans multiple experiments (including, for example, replicates), or when it is natural to examine different parts of the data using different views. The brushing concept is extended in CellProfiler Analyst for situations where multiple experiments are being simultaneously explored: when a point corresponding to a particular image is highlighted, all points corresponding to that experimental treatment condition can be highlighted, even if the data comes from multiple experiments that are being examined together. In the scatterplot in Figure 1b, for example, four data points are blue because one was originally selected and the user requested that replicates for that sample be highlighted.

### Investigating data

**Figure 3:**
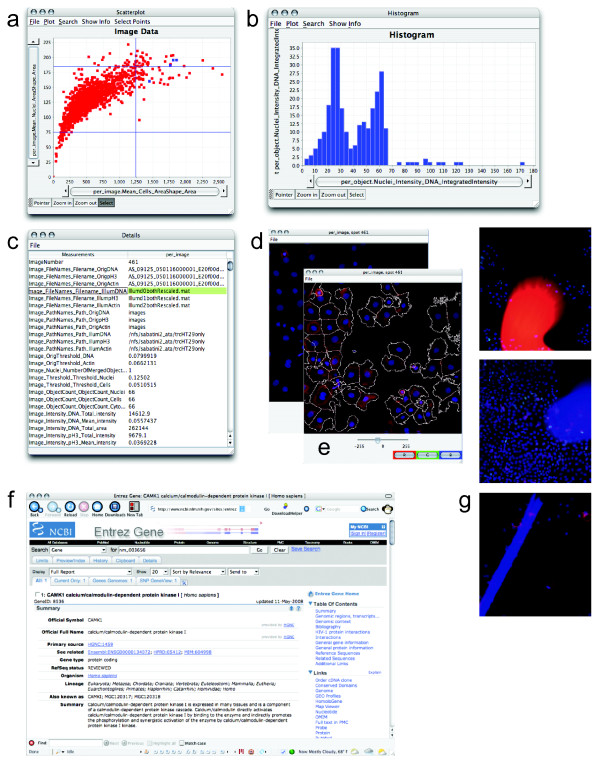
**Data points can be investigated**. The data points highlighted in blue in the scatterplot (a) represent replicates of a particular treatment condition with high mean cell area and nuclear area. To examine the cell cycle distribution of these samples, a DNA content histogram based on individual cells within those four images was plotted (b). For one of the four data points, the researcher displayed a table of all measurements in the database (c), the raw image (d), or with outlines overlaid (e). Each staining/channel (red, green, blue) can be toggled on or off to allow close examination of the relationships between them. Information from a public website describing the gene tested in one of the samples has been displayed (f) by clicking the data point. Outlier data points for certain measured features (e.g., high mean cell actin intensity, high mean cell area, high segmentation threshold, or percent of pixels that are saturated) can indicate images with severe artifacts (g) that should be excluded from analysis. These images can be identified by their aberrant measurements and excluded from further analysis by gating (i.e., selecting only a subset of data to be plotted and analyzed further).

Interesting data points or sets of data points can be investigated by drilling down into the data in several ways (Figure 3). For plots showing data points representing image measurements, a data point or set of data points can be selected and the original images that produced the data point can be displayed (Figure 3d). This can reveal artifacts in sample preparation or imaging, such as fluorescent test compounds, aggregates or overabundance of staining reagents, fibers, or debris (Figure 3g). These artifacts not only occlude actual cells in images but can also disrupt the proper identification and measurement of remaining cells in the image. For these and other reasons, images showing identification outlines resulting from image analysis (if available) can also be shown for selected data points (Figure 3e), to identify whether the identification of cells occurred properly. This is an important consideration given that no segmentation algorithms are flawless.

Additionally, a data point or set of data points can be selected and a plot of the measurements of *individual *cells that were present in those images can be displayed as a separate subplot. This allows, for example, a DNA content histogram indicating cell cycle distribution of the cell population to be displayed for a particular image or set of images of interest (Figure 2c and Figure 3b). To investigate the identity of interesting samples, a simple list of the treatment conditions that produced a set of data points can be displayed to get an overview (Figure 2d). For further investigation, web-based information about each image's treatment condition can be launched in an external web browser (Figure 3f), if web addresses associated with each sample are stored in the database. All available measurements and other information for a particular sample can be displayed in a simple table and saved as a comma-delimited text file for analysis in another software package (Figure 3c).

### Gating individual cell data to score complex phenotypes

**Figure 4:**
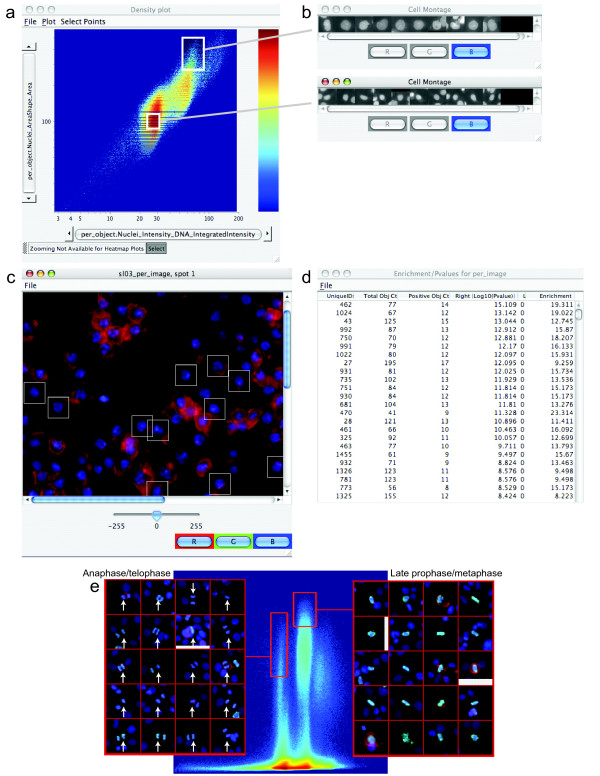
**Cell subpopulations can be identified, examined, and scored**. (a) On a density plot of individual cell data (log scale: X-axis = integrated intensity of nuclear DNA; Y-axis = nuclear area), two populations were gated (white boxes) and a random selection of cells within each subpopulation is shown in the montages on the right (b); all gated cells present in a particular sample can also be marked (c). Samples can be scored (d) for the number of gated cells and total cells in each sample, the enrichment of that percentage relative to the overall percentage of positive cells in the entire experiment (“Enrichment”; for example, the first image listed in the table has 19.311-fold more cells in the subpopulation than typical in the experiment overall), and the left- and right-tail log_10_ p-values (a measure of the statistical significance of the enrichment, based on the number of cells in the sample). (e) Gates for anaphase/telophase and late prophase/metaphase (data is plotted for all human HT29 cells in the experiment [7]. X-axis = integrated nuclear intensity of DNA, log scale; Y-axis = mean nuclear intensity of phospho-histone H3). Random cells falling within the gates are shown in the center of each 34 x 34 mm subimage.

Image-based data is tremendously valuable in that multiple single-cell measurements are available. Responses of individual cells to a treatment are usually inhomogeneous because of cell cycle variations or differences in protein levels due to memory or stochastic noise [15,16]. In many cases, a single measured feature (e.g., the total intensity of red stain within the nucleus) can be used to score individual cells and the only challenge is to identify a suitable threshold for scoring positive cells. This can be accomplished in CellProfiler Analyst using histograms of individual cell data. For complex phenotypes, several features of each cell may be required for effective scoring. In these cases, a density plot showing individual cells (Figure 4a) can be useful for identifying interesting cell subpopulations, by delineating a section of the plot (often called "gating"). Whether the gate contains the cells of interest can be tested using two features: the "Show Object Montage" feature to see what individual cells within the gate look like (Figure 4b), and the "Show Image" feature to see whether cells within a particular sample are appropriately marked as inside or outside the gate (Figure 4c). Once the final, desired subpopulation of cells is gated, the number of cells that fall within that subpopulation is calculated for each image, for further statistical analysis (Figure 4d). As an example, when DNA and phosphorylated Serine 10 of histone H3 are both stained, a simple two-feature gate in CellProfiler Analyst enables scoring mitotic subphases in human HT29 cells (Figure 4e). Many software systems perform image analysis on the fly during image acquisition; in such cases, a threshold value for a feature of interest must be chosen in advance to score the screen. By contrast, these tools in CellProfiler Analyst allow testing the efficacy of scoring based on different features and different measurement thresholds.

If more than two features are needed to score a phenotype, sequential gates can be used upon the cell data. This approach is applied as follows: (1) display the entire population of cells from an experiment in a density plot, (2) draw a gate around the data points representing potential cells of interest, (3) adjust the gate to include nearly all positive cells and exclude as many negative cells as possible, (4) plot the resulting gated subpopulation in a new density plot with two new measurement features as axes, (5) gate the subpopulation again based on these new features, and (6) calculate the percentage of each image's cells that fall within the final gate.

### Case study: mitotic subphase screen

#### Motivation

**Figure 5:**
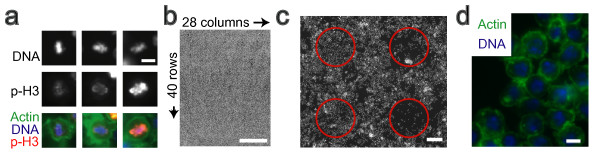
***Drosophila *cell microarrays**. (a) Phospho-histone H3 staining is often dim for metaphase nuclei (left and middle vs. right). Scale bar = 5 μm. (b) One cell array, DNA-stained and contrast-enhanced (5× lens). Scale bar = 5 mm. (c) Small section of (a), with circles denoting 4 spots on the array. Scale bar = 100 μm. (d) High-resolution image (40× lens) from within a spot of the array, stained with Hoechst (blue, DNA) and phalloidin (green, actin). Scale bar = 5 μm.

We wanted to test CellProfiler Analyst's ability to plot, explore, and filter individual cell data to identify subpopulations defined by several morphological features. We chose to identify *Drosophila melanogaster *Kc167 cells in telophase and metaphase of the cell cycle, using only a DNA stain. Identification of samples with perturbed cell cycle regulation is of clear importance to normal cell biology as well as cancer studies. Regulators of the cell cycle have been sought intensively for decades via traditional and high-throughput screens for changes in overall cell cycle distribution or for increased phospho-histone H3 staining, a marker of cells in late G2 and M phase (e.g., [17] and references therein). We reasoned that additional genes might exist which, when perturbed, yield increased numbers of metaphase- or telophase-stage nuclei without substantially affecting the overall mitotic index (phospho-histone H3 staining) or cell cycle distribution. While we were not aware of any positive controls with such a phenotype, we suspected such genes might have been previously overlooked because we noticed that not all metaphase nuclei stain brightly for phospho-histone H3 (Figure 5a), for unknown reasons. Identifying genes whose RNAi produces cells appearing to be in particular subphases of mitosis, regardless of concomitant phospho-histone H3 staining, would be a first step towards understanding these phenomena.

Several groups have tested automated methods for scoring mitotic subphases [18-20]; these studies were accomplished by computational tools tailored to the specific assay and often relied on multiple cellular stains. Machine learning methods have been explored by our own group and others [21-26] (and see Conclusions), but we also wanted to explore allowing the user to manually select a small number of features of known biological relevance, followed by sequential gating on those features. This would give the researcher full control over the features used in the scoring, and the scoring would be more readily transferable from one experiment to the next because a small number of features are selected. We therefore wanted to score mitotic subphases using a DNA stain only, using supervised selection of measurements followed by sequential gating on those measurements, in the context of a software package usable by a non-computer scientist.

#### Image scoring by sequential gating of individual cell data

We screened genes using *Drosophila *RNA interference living cell microarrays [27-29] to identify gene "knockdowns" that yield a disproportionate number of cells in two sub-phases of mitosis: metaphase and anaphase/telophase (referred to as telophase for simplicity). We created and analyzed 5 replicates of a *Drosophila *array, with 1120 spots of dsRNA on a single microscope slide (Figure 5b), including three replicate spots for each of 288 genes (mostly kinases and phosphatases), plus 256 negative control spots lacking dsRNA. Some phenotypes produced in these *Drosophila *Kc167 cells (e.g. cell death) are visible at low resolution (5× lens; Figure 5c), but to identify telophase and metaphase nuclei we collected individual high resolution images within each spot on each slide (40× lens; small portion of one image shown in Figure 5d).

**Figure 6:**
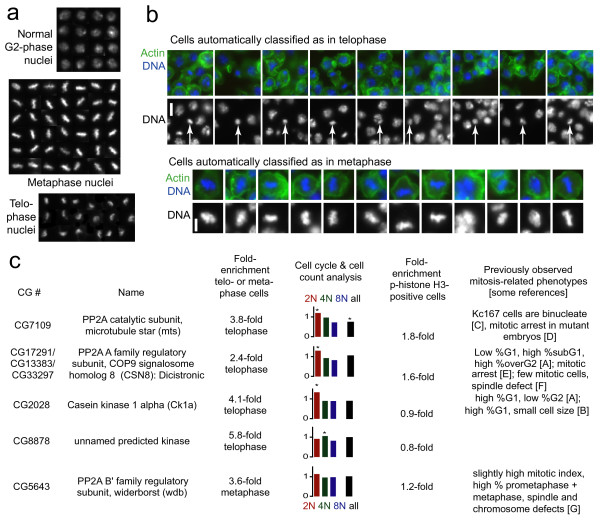
**Screen revealed RNA interference samples enriched in telophase- and metaphase-stage nuclei**. (a) Composite images of handpicked *Drosophila *Kc167 nuclei that were measured to guide feature selection and provide the starting point for developing the gates. Other nuclei at the edges of each sub-panel of the composite image were excluded from analysis. (b) A random sample of automatically scored nuclei from the cell microarrays. The scored cell is in the center of each panel unless the cell was near the edge of the image. Scale bars = 5 μm. (c) Genes whose dsRNAs significantly increase the proportion of cells in telophase (first four rows) or metaphase (last row). We excluded one gene (CG3245) that originally scored as a telophase hit because visual examination revealed that one image contained debris that disrupted proper analysis. Note that CG8878 should not be considered telophase-specific (see text). The fourth column shows the fold-change in the percentage of cells with each DNA content (2N, 4N, or 8N) and the fold-change in the total cell count overall, with significant differences from all genes tested on the array marked with a star. See Materials and Methods section for details on this image-based analysis of the cell cycle distribution. The fifth column indicates the fold-enrichment for the percentage of cells that were phospho-histone H3-positive, using the mean intensity of staining in the nucleus, based on the average of two replicates. References cited in the last column: A [17], B [42], C [43], D [44], E [45], F [20], G [36].

We began with the telophase phenotype. To determine which measured cellular features would be most effective for scoring, we handpicked representative telophase nuclei and normal G2-phase nuclei from random screening images and created image montages for these two classes (Figure 6a) using Adobe Photoshop. We used CellProfiler to measure nuclear features in these montage images, then exported the results to Excel and selected five features to use for sequential gating, based on a combination of biological intuition plus the quantitative ability of each feature to discriminate telophase from normal nuclei, using simple statistical tests in Excel. The selected features included DNA content, intensity, shape, and texture features (additional data file 1).

We then interactively developed sequential gates using density plots of these features in CellProfiler Analyst (see "Gating individual cell data to score complex phenotypes" section). To accomplish this task, CellProfiler was used to process the full set of screening images and load the resulting data into a database (2.8 million cells × 396 features/cell = 1.1 billion measurements total). This allowed us to display all individual cells in the experiment in an initial density plot with two of our selected features as axes, i.e., DNA content and size (area) of the nucleus. We drew an initial gate around the 2N DNA content peak and small nuclear area, and empirically refined the gate for telophase cells by examining images of the gated nuclei and adjusting the gate's boundaries accordingly. While automated approaches could certainly identify a boundary based on a researcher-provided training set, this manual approach allows the biologist to specifically assess many cells near the relevant boundaries. Once the appropriate gate was selected for the initial density plot, the subpopulation was transferred to a new density plot with two new features used as axes and the next gate was created, again finding the optimal parameters to distinguish telophase nuclei from all other nuclei. This procedure was repeated for the fifth, and final, selected feature. Once the final gate was refined, we applied the sequential gates to a new set of images and confirmed that their scoring was effective (Table 1 and Figure 6b), successfully differentiating telophase from other nuclei. In creating the gates, we tried to minimize the false positive rate while accepting a higher false negative rate (Table 1). We reasoned that true hits would have enough positives to overcome this intentionally stringent selection procedure. At this point, we applied the final sequential gates to all the cells in order to score the entire screen for the telophase phenotype. We found that the gates must typically be adjusted slightly between different replicate slides due to inter-experiment variability (e.g., staining intensity), although experiment-to-experiment normalization methods could be explored to reduce this effect.

**Table 1:** Accuracy of scoring the metaphase and telophase phenotype

	Metaphase		Telophase	
# positive cells (Observer 1)	35		25	
# positive cells (Observer 2)	40		38	
Mean # positive cells (Observers 1&2)	37.5		31.5	
# positive cells (CellProfiler)	16		20	
# cells called positive by CellProfiler but not by Observers	0		1	
# cells called positive by Observers but not by CellProfiler	21.5	(37.5-16)	11.5	(31.5-20)
false positive rate	0%	(0/16)	5%	(1/20)
false negative rate	57%	(21.5/37.5)	37%	(11.5/31.5)

Ten randomly selected images from the screen (~5000 cells total) were scored by CellProfiler Analyst and by two observers.

We separately performed the same procedure for the metaphase phenotype (using four features to distinguish metaphase nuclei from all other nuclei); a complete list of the 288 genes tested and their scores for telophase and metaphase is shown in additional data file 2.

#### Telophase analysis

**Figure 7:**
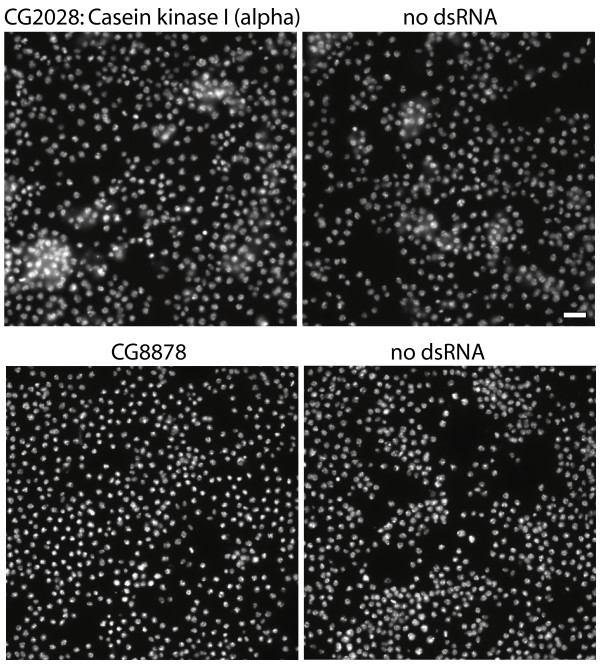
**Unusual phenotypes were found for telophase hits CG2028 and CG8878**. Top: CG2028 knockdown produces many telophase-like nuclei that appear less condensed than typical telophase nuclei but more condensed than interphase nuclei. Bottom: Nearly all nuclei from the CG8878 sample are more condensed than controls. See text for discussion of both genes. Scale bar = 20 μm.

Rank-ordering samples by the percentage of telophase nuclei revealed 4 gene knockdowns with a significant increase in telophase nuclei (Figure 6c, first 4 rows). Validating the approach, two of the genes are PP2A complex subunits that have been previously associated with mitosis: the PP2A-C catalytic subunit mts (CG7109/microtubule star) and a PP2A-A family regulatory subunit (CG17291/CG33297/CG13383, Note: dicistronic with CSN8). RNAi against both genes increased the percentage of cells that were phospho-histone H3-positive (Figure 6c, fifth column). A third hit, Ck1α (Casein kinase 1α/CG2028), has also previously been linked to mitosis (Figure 6c, last column). We noticed that its knockdown by RNAi produced nuclei whose chromatin appeared to be slightly less condensed than typical telophase nuclei (Figure 7), while still more condensed than interphase nuclei. The percentage of cells that were phospho-histone H3-positive was normal (Figure 6c, fifth column). Together, these observations suggest that this defect occurs in late-stage telophase/anaphase. The fourth hit was a predicted kinase with no functional annotation (CG8878). Visual inspection revealed that nearly all nuclei in these samples appeared brighter and more compact than controls, a subtle but reproducible effect (Figure 7). This understandably resulted in more of the 2N nuclei being counted as having telophase-like morphology. We found that these cells were not enriched for phospho-histone H3-positivity (Figure 6c, fifth column); without further experimentation, it is unclear whether this is a true late-stage mitotic phenotype or rather a condensed nuclei phenotype.

#### Metaphase analysis

**Figure 8:**
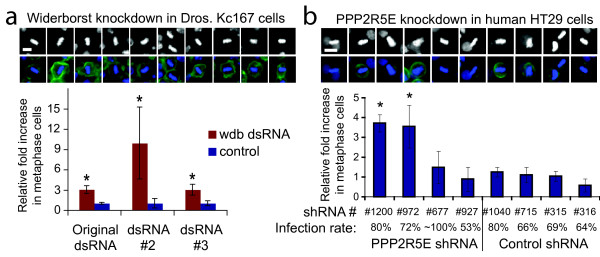
**The widerborst RNA interference metaphase arrest phenotype is confirmed in *Drosophila *and human cells**. (a) A sampling of metaphase nuclei produced by widerborst knockdown in *Drosophila *Kc167 cells (top). Scale bar = 5 μm. Quantitative confirmation of the increased percentage of metaphase nuclei, as scored by two blinded observers (bottom). Bars are starred if p < 0.05. Error bars = SEM. Controls are nearby spots lacking dsRNA. (b) A sampling of metaphase nuclei produced by PPP2R5E knockdown in human HT29 cells (top). Scale bar = 10 μm. Quantitative confirmation (bottom). Bars are starred if p < 0.001. Error bars = SEM. The infection rate is the ratio of the number of cells with/without puromycin, the selection agent. The control shRNA is FLJ25006 (NMid = NM_144610).

Interestingly, the only metaphase hit in this screen (Figure 6c, last row) is the B'/B56 subfamily regulatory subunit of PP2A (CG5643/widerborst), which at the time of our screen had not been linked to cell cycle regulation. The percentage of cells that were phospho-histone H3-positive was not much higher than normal (Figure 6c, fifth column). We confirmed by eye the metaphase-inducing phenotype of widerborst knockdown in the original images and in separate experiments with two other dsRNAs, including one that was non-overlapping with the original (Figure 8a). Widerborst is an essential gene involved in planar cell polarization [30] and apoptosis [31,32]. Notably, in other contexts (circadian clock protein cycling [33] and sensory organ development [34]) widerborst is indirectly linked to the B/PR55 subfamily member twins/aar, which is itself known to be required for metaphase to anaphase transition [35]. Our work therefore confirms, with non-overlapping dsRNAs, a recently reported cell cycle regulation role for widerborst [36] and together indicates that it is unlikely this phenotype is due to off-target effects [37,38].

The closest human homolog of widerborst is PPP2R5E, the epsilon isoform of a subfamily of PP2R5 (a.k.a. B'/PR61/B56) regulatory subunits of the PP2A complex. As yet, no particular function has been associated with PPP2R5E. We wondered whether PPP2R5E might be a B' regulatory subunit that modulates the known role for PP2A in mitosis, given our finding of its homolog widerborst's role in *Drosophila*. PPP2R5E knockdown did not increase the mitotic index significantly in recent RNA interference screens for increased phospho-histone H3 [7,39,40]. However, when we scored these same PPP2R5E-knockdown images for metaphase morphology, rather than phospho-histone H3 levels, we discovered a metaphase-arrest phenotype for PPP2R5E knockdown, confirmed by two different shRNAs (Figure 8b), consistent with the phenotype seen for widerborst in *Drosophila*. Whether widerborst/PPP2R5E are themselves required for metaphase-to-anaphase transition or whether their depletion causes the phenotype by specifically disrupting the stoichiometry of the relevant PP2A complex remains to be determined. Recent findings that PPP2R5E localizes to centromeres and that the B' subfamily of regulatory subunits are required for proper meiotic sister chromatid separation in fission and budding yeast [41] support the idea that this family of subunits is indeed important for proper chromatin dynamics during cell division.

## Conclusion

We have described here a software system for exploration and analysis of large, hierarchical, multi-dimensional data sets. While it is compatible with any type of data (e.g., players on teams, trees within forests), it is particularly capable of high-end exploration and analysis of measured features from high-throughput image-based screens for both quality control and identifying hits in a screen. Researchers are welcome to download the Java source code and add new types of plots and analysis tools (e.g., for normalizing screen data [9,10]) to the system.

We have demonstrated the utility of this software for interactive data exploration and analysis – especially for intentionally selecting cells with particular measurement values in order to score complex visual phenotypes. Of course, often the features that successfully specify a particular phenotype are either unknown or so numerous as to make the sequential plotting shown here impractical, and choosing decision boundaries empirically may not be optimal to score the phenotype. For these reasons, we recently added machine-learning methodology to CellProfiler Analyst (TRJ, AEC, DMS, PG, unpublished data). Nonetheless, the complete control over features and thresholds offered by sequential gating is quite useful in some cases. Often a researcher needs to ignore certain features of positive control cells (for example, when a positive control treatment has pleiotropic effects on cells) and emphasize other, better-understood cellular features. Interactive observation of the original cellular images while making gating decisions to define a phenotype also leverages the biologist's intuition about a phenotype. Within the same open-source software infrastructure, both approaches (sequential gating and machine learning) can now be applied to large-scale imaging screens.

## Availability and requirements

* Project name: CellProfiler Analyst

* Project home page: 

* Operating systems: Platform independent (Mac, Windows, and Unix)

* Programming language: Java

* Other requirements: Java 1.4.2 or greater. For full functionality, CellProfiler Analyst requires Java 1.5.0_6 or greater, Python version 2.5 or greater  and the NumPy Python package .

* License: GNU GENERAL PUBLIC LICENSE, Version 2

* No additional restrictions to use by non-academics

## Methods

### Software

CellProfiler Analyst can be downloaded for Mac, Windows, and Unix operating systems from the CellProfiler Project website , where it is distributed under an open-source license (GNU General Public License, version 2). An archived version is also available as additional data file 3, submitted with this article. The Examples page of the website provides demonstration movies showing the software in use, an example database and images, and links to an online forum where questions about the software are answered.

CellProfiler Analyst is designed to explore and analyze any MySQL database of image-based screening data that follows a simple format: at least one image table with rows corresponding to images and columns of image data (examples of columns are: the name of the treatment condition, total intensity of the entire image, mean cell area averaged over all cells in the image, path to the original image), and at least one object table with rows corresponding to objects (e.g., cells) and columns of object data (examples of columns are: area of the cell, intensity of DNA stain in the nucleus, location of the cell in the original image – the latter being important for viewing individual cells during exploration). This data format is automatically produced if images are analyzed with CellProfiler open-source cell image analysis software [4], using its ExportToDatabase module. The data should be normalized for plate-to-plate or spatial-layout variations prior to exploration in CellProfiler Analyst. While the software is designed to access remote databases because typical data sets are far too large to be stored in physical memory, the "Make Local Object Table" option allows particularly relevant measurements to be stored locally in memory to speed analysis while still allowing access to the full dataset in the remote database.

### Cell culture

We prepared *Drosophila *Kc167 cells as previously described [29]. In brief, cells were grown on living cell microarrays with spots of double stranded RNA for 3 days. For confirmation of phenotypes in Drosophila, we grew cells on plain slides for 3 days, after being pre-treated with dsRNA for 2 days. We used images of human HT29 cells as previously described [7].

### Statistical analysis

For the screen of the metaphase and telophase phenotypes, each gene was tested in three replicate spots on five independently prepared cell array slides, and the results for all genes are shown in Additional file 2. Because the three replicate spots were near each other, cell counts for the groups of three were accumulated and not treated as independent samples. A p-value for each gene on each of the five slides was calculated based on the number of metaphase nuclei found and the number of cells total, relative to the average percentage of metaphase nuclei on the entire slide (i.e., as a Bernoulli random variable). To add stringency, we report results for second- and third-strongest scoring replicates only (shown on two separate sheets of Additional file 2). We required that two or three of the five scores were above a threshold that results in a combined p-value below 0.01. For Bonferroni-adjusted p-values from single experiments, these thresholds are 0.6 for two experiments (out of 5), and 5.2 for three (out of 5). De-enriched samples are listed with a p-value of 1 and samples with a p-value of 1 are ordered by enrichment. "Enrichment" is the fold-enrichment of the sample relative to all the samples.

In the bar charts in the fourth column of Figure 6c, we statistically analyzed the cell cycle distribution and cell count for the screens' hits. To do this, we first gathered DNA content data (i.e., integrated nuclear DNA intensity) from the database for all cells on the slides where the hits occurred. Then, to normalize for illumination and staining variation between slides and between images, the DNA content measurements were log2-transformed and shifted so that the mode of the DNA content for each image (calculated by binning the log2-transformed DNA into 50 bins) was equal to 1. Based on this normalized log2(DNA intensity), cells were then counted as 2N, 4N, and 8N as follows:

[-0.5, 0.5) was categorized as "2N"

[0.5, 1.5) was categorized as " 4N"

[1.5, 2.5) was categorized as " 8N", although this includes ~6N to ~11N

Using the resulting cell counts for each subpopulation (2N, 4N, 8N), we calculated p-values as follows: first, subpopulation counts were converted to fractions for each image by dividing the subpopulation counts by the total number of cells in the image (taken as the sum of the 2N, 4N, and 8N subpopulations). Each fraction was then normalized by the median fraction for that subpopulation on that slide, to account for any per-slide biases in cell-cycle distribution. These normalized fractions were averaged across replicate samples for each gene. Lastly, these averaged normalized fractions were used to calculate p-values for each subpopulation in a 10,000-trial permutation test (where labels were permuted within slides, but not between slides, to ensure that the same number of images was taken from the slide as in the experiment). Cell-count p-values were calculated similarly: the total number of cells in each image was normalized by the median per-image cell count on that slide, to prevent biases for more densely populated slides, and a permutation test was performed on the average normalized cell-count. For cell cycle and cell count, p-values were Bonferroni-corrected for 20 experiments (5 genes examined for 4 populations: 2N, 4N, 8N, and count).

## Authors' contributions

The software project was conceived, designed, and developed by TRJ, AEC, PG, DMS, and IHK. IHK wrote most of the software's code, RAL and DBW prepared the cell microarrays and *Drosophila *follow-up experiments, and TRJ and AP made adjustments to the software. AEC and TRJ used the software to perform the screen, and together analyzed and interpreted the data. The manuscript was prepared by AEC, then revised and approved by all authors.

## Supplementary Material

Additional file 1 **Features tested to identify the best for constructing the metaphase and telophase gates.** Click here for file

Additional file 2 **List of all genes tested, with scores for metaphase and telophase nuclei enrichment.** Click here for file

Additional file 3 **Source code for CellProfiler Analyst** (most recent version is available at ). Additional File 3 must be uncompressed. Doubleclicking the file often activates the computer's native decompression software; otherwise download free software online (e.g., StuffIt Expander) for this purpose. Click here for file
