# Supplementary figures and images for: CellProfiler Analyst: data exploration and analysis software for complex image-based screens
Source: BMC Bioinformatics. 2008 Nov 15;9:482. doi: 10.1186/1471-2105-9-482 (PMC2614436; doi:10.1186/1471-2105-9-482)

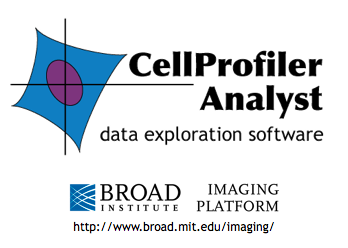

Supplement: Additional file 3 — Source code for CellProfiler Analyst (most recent version is available at ). Additional File 3 must be uncompressed. Doubleclicking the file often activates the computer's native decompression software; otherwise download free software online (e.g., StuffIt Expander) for this purpose. [file 1471-2105-9-482-S3.zip › CPA_src/jCPAnalyst/SplashScreen.png]

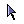

Supplement: Additional file 3 — Source code for CellProfiler Analyst (most recent version is available at ). Additional File 3 must be uncompressed. Doubleclicking the file often activates the computer's native decompression software; otherwise download free software online (e.g., StuffIt Expander) for this purpose. [file 1471-2105-9-482-S3.zip › CPA_src/jCPAnalyst/Visualize/JFreeChartExt/toolbarGraphics/Pointer24.gif]

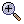

Supplement: Additional file 3 — Source code for CellProfiler Analyst (most recent version is available at ). Additional File 3 must be uncompressed. Doubleclicking the file often activates the computer's native decompression software; otherwise download free software online (e.g., StuffIt Expander) for this purpose. [file 1471-2105-9-482-S3.zip › CPA_src/jCPAnalyst/Visualize/JFreeChartExt/toolbarGraphics/ZoomIn24.gif]

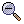

Supplement: Additional file 3 — Source code for CellProfiler Analyst (most recent version is available at ). Additional File 3 must be uncompressed. Doubleclicking the file often activates the computer's native decompression software; otherwise download free software online (e.g., StuffIt Expander) for this purpose. [file 1471-2105-9-482-S3.zip › CPA_src/jCPAnalyst/Visualize/JFreeChartExt/toolbarGraphics/ZoomOut24.gif]
